# Statoviruses, A novel taxon of RNA viruses present in the gastrointestinal tracts of diverse mammals

**DOI:** 10.1016/j.virol.2017.01.010

**Published:** 2017-01-30

**Authors:** Andrew B. Janowski, Siddharth R. Krishnamurthy, Efrem S. Lim, Guoyan Zhao, Jason M. Brenchley, Dan H. Barouch, Chrissie Thakwalakwa, Mark J. Manary, Lori R. Holtz, David Wang

**Affiliations:** a Department of Pediatrics, Washington University School of Medicine, St Louis, MO, USA; b Department of Molecular Microbiology and Pathology and Immunology, Washington University School of Medicine, St Louis, MO, USA; c Lab of Parasitic Diseases, National Institute of Allergy and Infectious Diseases, National Institutes of Health, Bethesda, MD, USA; d Center for Virology and Vaccine Research Beth Israel Deaconess Medical Center, Boston, MA, USA; e Ragon Institute of MGH, MIT, and Harvard, Boston, MA, USA; f Department of Community Health, College of Medicine, University of Malawi, Blantyre 3, Malawi

**Keywords:** Statoviruses, Tombusviridae, Flaviviridae, Viral discovery, Virome

## Abstract

Next-generation sequencing has expanded our understanding of the viral populations that constitute the mammalian virome. We describe a novel taxon of viruses named Statoviruses, for **St**ool **a**ssociated **To**mbus-like viruses, present in multiple metagenomic datasets. These viruses define a novel clade that is phylogenetically related to the RNA virus families *Tombusviridae* and *Flaviviridae.* Five distinct statovirus types were identified in human, macaque, mouse, and cow gastrointestinal tract samples. The prototype genome, statovirus A, was frequently identified in macaque stool samples from multiple geographically distinct cohorts. Another genome, statovirus C1, was discovered in a stool sample from a human child with fever, cough, and rash. Further experimental data will clarify whether these viruses are infectious to mammals or if they originate from another source present in the mammalian gastrointestinal tract.

## 1. Introduction

Unbiased next-generation sequencing (NGS) techniques have demonstrated the remarkable abundance and diversity of viruses that populate mammalian organisms (Virgin, 2014). Viruses known to infect eukaryotes, bacteria, archaea, and novel viruses of unknown host-tropism comprise the mammalian virome (Virgin, 2014; Handley et al., 2012; De Vlaminck et al., 2013; Phan et al., 2011; Li et al., 2010; Krishnamurthy et al., 2016). Dynamic changes in the composition of the virome have been observed in humans and macaques (Krishnamurthy et al., 2016; Lim et al., 2015; Reyes et al., 2010) as well as a dependency on host immune status. In humans after solid organ transplant, the abundance of anelloviruses significantly increases after the initiation of immunosuppression (De Vlaminck et al., 2013). Furthermore, acquired immunodeficiency syndromes caused by human immunodeficiency virus (HIV) and simian immunodeficiency virus (SIV) infection are characterized by expansion of enteric eukaryote-infecting viral families, including *Adenoviridae* and *Picornaviridae (*Handley et al., 2012; Handley et al., 2016; Monaco et al., 2016; Li et al., 2013).

The virome also includes viruses that are assumed to be inert in regards to interaction with the mammalian host. For example, some viruses detected from the gastrointestinal tract are thought to be simply “passengers” due to dietary exposure or environmental contamination (Balique et al., 2015; Zhang et al., 2006). Most commonly these are plant- or insect-infecting viruses; however, some recent studies have demonstrated that such viruses can interact with and affect the host. Acanthocystis turfacea chlorella virus 1, a member of the double stranded DNA virus *Phycodnaviridae* family, whose known members primarily infect green algae, has been identified in human oropharyngeal samples, replicates in human macrophages, and is associated with decreased cognitive function in humans and mice (Yolken et al., 2014; Petro et al., 2015). As another example, Pepper mild mottle virus, a single stranded positive sense RNA plant virus of the family *Virgaviridae,* is frequently detected in human stool samples and has been associated with symptoms of fever, abdominal pain, and pruritus, as well as a human serological response (Colson et al., 2010).

Many novel viruses within the virome have been identified but for most, their effects on the host and other members of the microbiome remain to be elucidated (Handley et al., 2012; Li et al., 2010; Krishnamurthy et al., 2016). For taxonomical analysis of members of the virome, classification of the novel viruses frequently relies upon sequence alignment to known reference viruses (Zhao et al., 2013; Sharma et al., 2015; Cantalupo et al., 2011). Assumptions regarding tropism and potential pathogenicity are often inferred from that of the most similar reference virus. While this may be reasonable for novel viruses that share high similarity to the reference virus, this may be problematic and lead to inaccurate inferences when viral sequences are highly divergent from the reference. All RNA viruses except retro-viruses encode a RNA-dependent RNA polymerase (RdRp) for replication (Holland et al., 1982; Bruenn, 2003; Koonin, 1991). Phylogenetic superfamilies using the RdRp domain have been identified with one of the earliest analyses by Eugene Koonin in 1991, but these taxonomical units do not correlate with tropism (Koonin, 1991; Holmes, 2009). For example, one RdRp superfamily domain, Superfamily II (also known as RdRp 3 on NCBI Conserved Domain Database [CDD] and as Pfam domain PF00998), contains members of the plant infecting viral family *Tombusviridae* and the vertebrate/arthropod infecting viral family *Flaviviridae* (Finn et al., 2016; Marchler-Bauer et al., 2015).

We describe the identification of multiple members of a novel taxon of viruses detected from the gastrointestinal tract of mammals. These viruses, which have a distinctive genome organization, share phylogenetic similarity to members of the RdRp Superfamily II domain, and thus are related to both viruses that infect animals and plants. Sequencing reads from these viruses were detected in 25% of the primate stool samples examined demonstrating that they are frequent constituents of primate viromes. Furthermore, additional divergent members of this taxon were also identified in public sequencing datasets, demonstrating more widespread infection by this group of viruses.

## 2. Materials and methods

### 2.1. Macaque stool cohorts

Metagenomic data from samples from three previously published macaque cohorts were analyzed (Handley et al., 2012, 2016; Barouch et al., 2015; Klatt et al., 2013). The cohorts consisted of SIV infected macaque samples from study sites within the United States, including stool samples from *Macaca nemestrina* (pig-tailed macaques) housed at the National Institutes of Health (NIH) in Bethesda, Maryland, and *Macaca mulatta* (rhesus macaques) at the New England Primate Research Center (NEPRC) in Southborough, Massachusetts, Bioqual located in Rockville, Maryland, and from the Tulane National Primate Research Center (TNPRC) located in Covington, Louisiana (Handley et al., 2012, 2016; Barouch et al., 2015; Klatt et al., 2013).

*Cohort #*1. The first study cohort analyzed the effect of probiotics and antivirals on pigtail macaques infected with SIV that were housed at the NIH (Klatt et al., 2013). This cohort was comprised of a total of 11 macaques with a total of 25 residual stool samples that were available for further analysis.
*Cohort #2.* The second study cohort included SIV or mock infected rhesus macaques that resided at NEPRC and TNPRC sites and included a total of 120 samples representing 86 individual macaques (Handley et al., 2012).
*Cohort #3*. Rhesus macaques housed at NEPRC and Bioqual composed the third study cohort that assessed efficacy of SIV vaccine formulations (Handley et al., 2016; Barouch et al., 2015). A total of 71 stool samples from 36 macaques were available for analysis (Handley et al., 2016; Barouch et al., 2015).

### 2.2. Cohort of human subjects with environmental enteropathy

Stool samples were collected as a part of a previously published study of Malawian twins discordant for malnutrition (Reyes et al., 2015; Smith et al., 2013). Within this cohort, 24 samples were selected for evidence of environmental enteropathy by an elevated urine lactulose:mannitol ratio, with 24 age matched controls without an elevated urine lactulose:mannitol ratio (Smith et al., 2013; Denno et al., 2014). Each child also had multiple additional longitudinal stool samples that were collected as a part of this cohort that were available for further analysis. Home environment, recent symptoms of illness, height and weight measurements, and a general physical examination were documented on all of the subjects.

### 2.3. Sample preparation and next generation sequencing analysis

All stool samples were diluted 1:6 in phosphate buffered saline (PBS), filtered through a 0.45 μm filter, with total nucleic acid (TNA) isolated by automated extraction with COBAS Ampliprep instrument (Roche) (Handley et al., 2012, 2016; Barouch et al., 2015). TNA were then reverse transcribed, and randomly PCR amplified as previously described (Handley et al., 2012, 2016; Barouch et al., 2015). Macaque cohorts 1 and 2 were then sequenced using the 454 GS-FLX Titanium (Roche) platform while the macaque cohort 3 and the human environmental enteropathy cohort were sequenced by the MiSeq Platform (Illumina) (Handley et al., 2012, 2016; Barouch et al., 2015). The sequencing results of cohort 1 have been deposited as NCBI Bioproject accession number PRJNA353872. Cohort 2 was deposited in MG-RAST as projects 1449, 1451, 1452, and cohort 3 was deposited in the European Nucleotide Archive as project number PRJEB9503. The human cohort of environmental enteropathy has been deposited as NCBI Bioproject PRJNA353874.

Sequencing results from the 454 GS-FLX Titanium platform were processed through a custom viral discovery algorithm, VirusHunter, which queries reads using BLASTn and BLASTx against the NCBI NT and NR database (Zhao et al., 2013). The sequencing runs from the MiSeq platform were processed through another custom pipeline, VirusSeeker that utilizes BLASTn and BLASTx against a customized viral database which can be downloaded from http://pathology.wustl.edu/virusseeker/VirusSeeker_Virome/VirusSeeker_Virome_index.htm (Lim et al., 2015; Handley et al., 2016; Cimino et al., 2014). Sequencing reads were assembled into contiguous sequences (contigs) by IDBA using default parameters (Peng et al., 2012).

### 2.4. Computational screening of existing sequencing datasets

We queried our own data sets from the macaque and human cohorts with command line BLASTn and tBLASTn, using the novel contigs as the query sequence. To identify public datasets from the NCBI SRA database for analysis, we downloaded datasets that resulted from a search using the terms of “stool”, “feces”, “sewage”, “waste-water”, freshwater”, “ocean”, “viromes”, “metatranscriptome”, “mammal”, “plant”, or “fungus” (Krishnamurthy et al., 2016). We only included datasets in which RNA was annotated as being sequenced, for a final total of 5,457 SRA datasets (Krishnamurthy et al., 2016). The amino acid sequences of open reading frame 1 (ORF1) and ORF2 of statoviruses were queried against these datasets using the command line tBLASTn. Samples in which more than five reads were present with alignment bitscores > 50 were selected for de novo assembly using the entire datasets from that sample, using IDBA with default parameters (Peng et al., 2012).

### 2.5. Identification of RdRp superfamily and coat protein domains

The ORFs of statoviruses were annotated by ORF Finder (NCBI) using the first in-frame ATG sequence as the start codon of the ORF. We determined the pairwise identity of the ORFs of statoviruses by generating a multiple sequence alignment using Clustal Omega (Sievers et al., 2011). Pairwise identities of the amino acid sequences of the ORFs of each of the five representative statovirus genomes were calculated. We queried the amino acid sequence of the five representative statovirus genomes (statovirus A1-E1) against the Pfam database using Hmmscan (Finn et al., 2011) to define the RdRp superfamily to which statoviruses belong. The RdRp domain of each statovirus genome was annotated by using Hmmscan (Finn et al., 2011) and the coat protein domain by Phyre2 (Kelley et al., 2015).

### 2.6. Generation of sequence alignments and phylogenetic trees

To infer the phylogenetic relationship of statoviruses to other RNA viruses, amino acid sequences of the putative statovirus RdRp domains and the consensus RdRp Superfamily II domain sequence from CDD were used to query the NCBI nr database (accessed on December 2, 2016) using DELTA-BLAST with a cutoff of bit score ≥55 and e-value ≤5e-7 (Finn et al., 2016; Marchler-Bauer et al., 2015; Boratyn et al., 2012). The search identified members of four viral families *(Tombusviridae*, *Luteoviridae*, *Carmotetraviridae* and *Flaviviridae)* and unclassified viruses. We curated a list of virus sequences with representative species of each genus within the four viral families *(Tombusviridae*, *Luteoviridae*, *Carmotetraviridae* and *Flaviviridae)*, all unclassified virus subjects from the above BLAST searches, and representative viral sequences from two recent publications that were absent from our NCBI nr database search (Shi et al., 2016, 2015). Alignments were performed using MUSCLE (Edgar, 2004) and was trimmed with trimAl (Capella-Gutierrez et al., 2009). The Wenling shark virus sequence (YP_009179227.1) was unalignable and thus excluded. This yielded an RdRp amino acid multiple sequence alignment of 77 sequences that included seven statovirus sequences (Supplementary data s1). The best-fit model of protein evolution was determined by ProtTest v3.4 (Darriba et al., 2011). Bayesian Markov chain Monte Carlo (MCMC) inference (LG + I + G + F) was performed with BEAST v1.8.3 (Drummond et al., 2012). Analyses were performed with a chain length of 10 million states (sampled every 1,000 iterations) under an uncorrelated relaxed clock (lognormal distribution) and Yule prior. Convergence and mixing was assessed with Tracer (v1.5) (Rambaut et al., 2014) and the maximum clade credibility tree was generated after a 25% burn-in period. Maximum likelihood (ML) analyses (LG + I + G+ F) were performed with PhyML (v3.0) with a discrete γ distribution of 4 rate categories (Guindon and Gascuel, 2003). Support for ML trees was assessed by 1,000 nonparametric bootstraps.

Nucleotide sequences of the statovirus A PCR amplicons and representative contigs were aligned by Muscle in Mega7 (Kumar et al., 2016). Maximum likelihood phylogenetic trees (GTR + G + I, best fitting model selected by JmodelTest) were constructed in Mega7 with 100 bootstraps (Kumar et al., 2016; Darriba et al., 2012).

### 2.7. RT-PCR, 3′ RACE, and Sanger sequencing

Reverse transcription polymerase chain reaction (RT-PCR) was completed with the OneStep kit (Qiagen) using the manufacturer’s instructions with primers and annealing conditions presented in Table 1 for confirmation of contigs in total nucleic acid samples. For each reaction, 10 pmol of primers were used, except for primer set AJ0057/AJ0059 in which 50 pmol per reaction were used. Reaction conditions included: 50 °C for 45 min, 95 °C for 15 min, then 40 cycles of 94 °C for 30 s, annealing temperature as in Table 1 for 30 s, 72 °C for 1 min, followed by a final extension phase at 72 °C for ten minutes. PCR products were then cloned into pCR4 TOPO plasmids (Invitrogen) and were then Sanger sequenced. 3′ Rapid amplification of ends (RACE) was completed with poly-adenylation of RNA as per the manufacturer’s instructions (ThermoFisher), and then 3′ RACE primers were used as in Table 1 for generation of PCR products (Felix et al., 2011). These products were then cloned into pCR4 TOPO plasmids, and Sanger sequenced. All confirmatory sequencing of the statovirus A2 and C1 genomes were completed with three replicates.

## 3. Results

### 3.1. Detection of a novel viral sequence

**Fig. 1:**
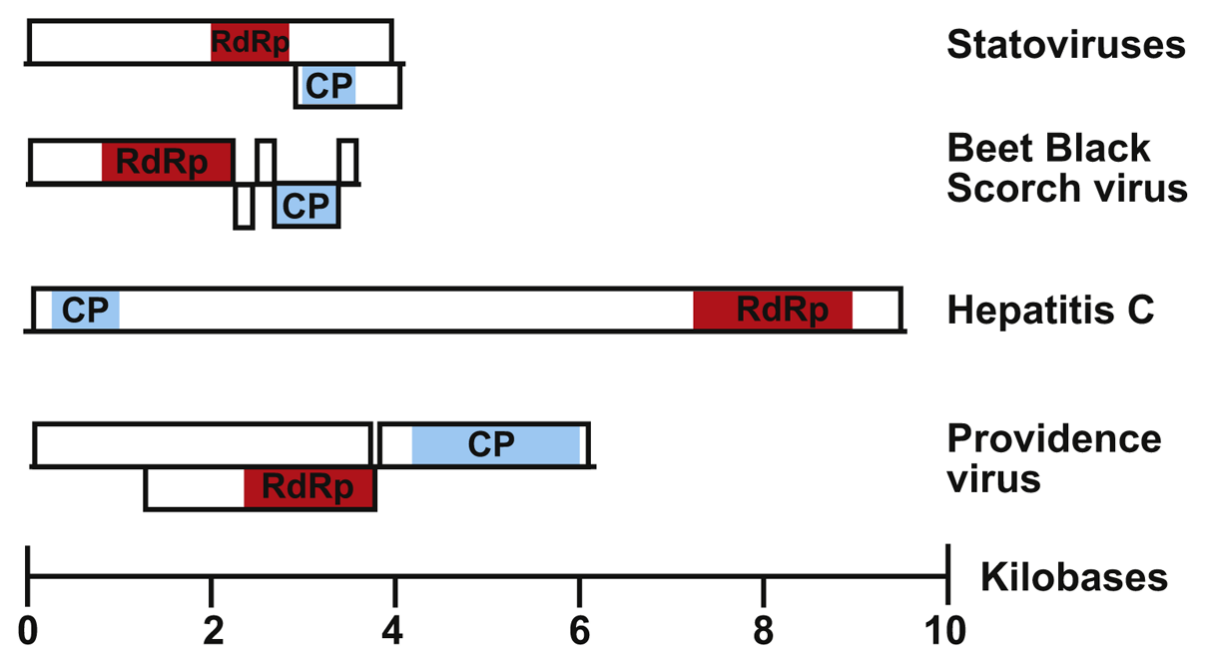
Genome organization with open reading frames of the representative viruses that contain RNA-dependent RNA polymerase (RdRp) Superfamily II domains, including statoviruses, beet black scorch virus, hepatitis C, and providence virus. Hepatitis C contains a single ORF that upon translation produces a polypeptide that contains both the RdRp domain and coat protein domain. The annotated protein domains for each virus are highlighted, the RdRp domain in red and coat protein (CP) domain in blue. Size in kilobases is depicted on the x-axis. (For interpretation of the references to color in this figure legend, the reader is referred to the web version of this article.)

As part of ongoing efforts to analyze metagenomic datasets for novel viruses, we initially identified sequence reads from one stool sample from macaque cohort 1 that shared 30–35% sequence identity by BLASTx to the RdRp of Beet black scorch virus, a member of the single stranded, positive sense RNA plant-infecting viral family *Tombusviridae*. The sample originated from a macaque stool specimen prior to infection with SIV. We hypothesized that these reads originated from a novel virus because of the divergence in the amino acid sequences between the reads and the most similar virus. Following de novo assembly of reads from this sample, a 4,158 nucleotide (nt) contig was assembled with average coverage of > 7x. Two ORFs were identified including ORF1, a larger ORF that spans most of the genome and contains the RdRp domain and ORF2, a smaller ORF that has an out-of-phase overlap with ORF1 (Fig. 1). This organization differed from the genome organization of beet black scorch virus (Fig. 1). Given the origin of the sample and identifiable sequence alignment to tombusviruses, we adopted the name “Statovirus” for Stool Associated Tombus-like virus, and this particular contig was designated “statovirus A1” (NCBI GenBank accession number KX792976). Using the statovirus A1 contig as the query sequence for BLASTn, reads with high nucleotide similarity (> 75%) were identified from a different macaque in cohort 1 that had three stool specimens positive for statovirus A1.

In total from the three macaque cohorts, reads with > 75% nucleotide identity to statovirus A1 were present in 55 out of 216 (25%) samples. We assembled contigs greater than 500 nt in length from 32 macaque stool specimens in cohorts 1–3. To validate the assembly, we arbitrarily selected a single sample from cohort 2 for Sanger sequencing using primers generated from regions with high conservation between all contigs. A total of 4,136 nucleotides were Sanger sequenced to ≥3x coverage from this sample, and this contig was named “statovirus A2” (NCBI GenBank accession number KX792977). We compared the assembled 454 GS-FLX Titanium platform sequencing contigs from this sample to the Sanger sequenced genome and there was 99.23% sequence identity between the two sequencing modalities. We further defined the 3′ terminus of the contig using 3′ RACE with three replicates. The nucleotide sequence of the statovirus A2 contig shared 87% nucleotide identity by BLASTn to A1. We also selected the longest contig to represent cohort 3 and named it “statovirus A3” (3,985 nt; > 27x coverage; NCBI GenBank accession number KX792978).

We compared the sequence identity between the ORFs of statovirus A1–3 using a multiple sequence alignment. In the region of ORF1 that did not overlap with ORF2, there was 86–94% pairwise nucleotide identity, while nucleotide sequences within ORF2 had 87–95% pairwise nucleotide identity. We also aligned the 3′ untranslated region of the contigs that contained the 3′ end of the genome, which include statovirus A1 and A2, and used the statovirus A2 genome to denote the terminal nucleotides. A total of 41/46 (89%) of the nucleotides downstream of the stop codon of ORF2 were conserved, including the terminal 41 out of 42 (98%) nucleotides at the 3′ end.

### 3.2. Identification of distantly related statovirus contigs

**Fig. 2:**
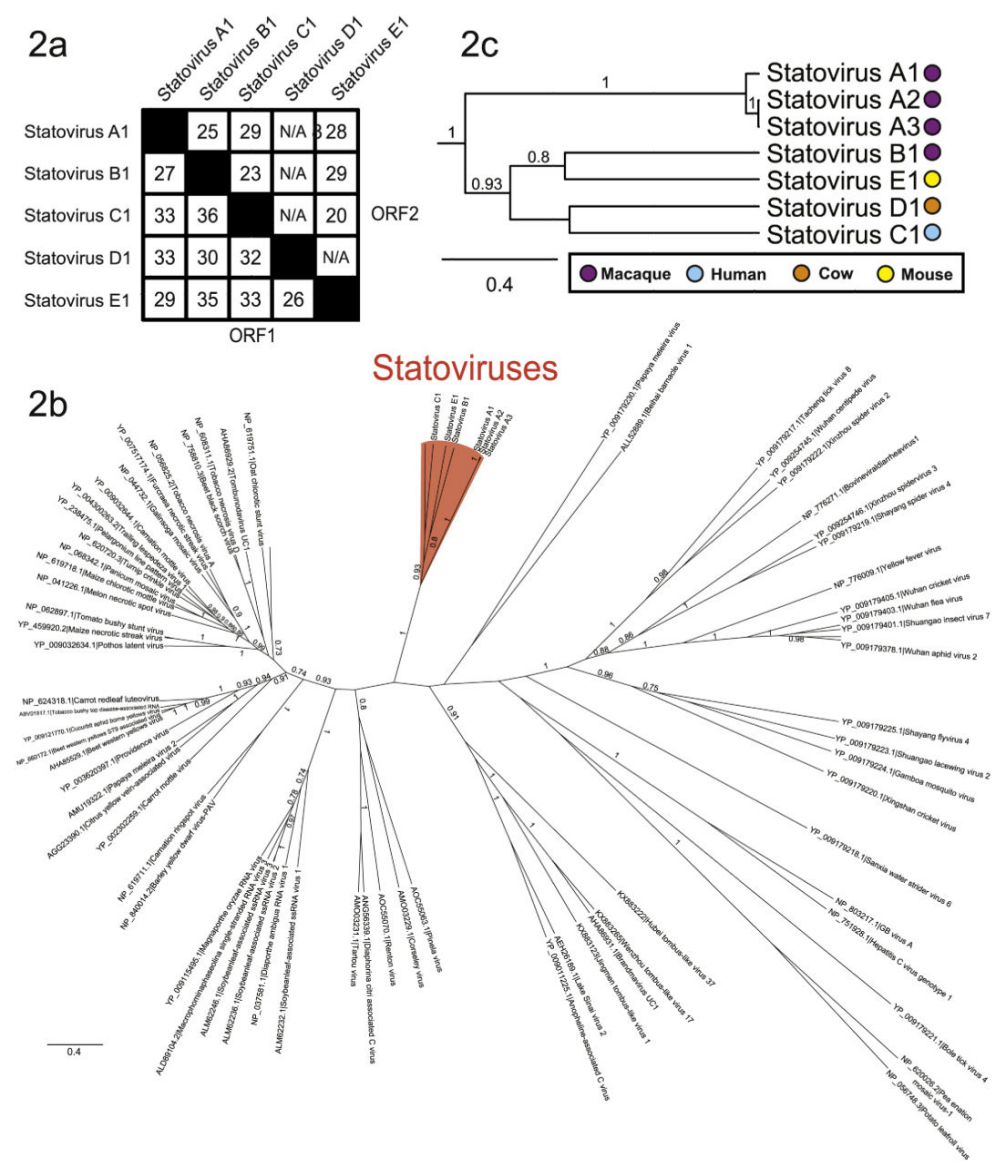
a Pairwise amino acid identities of statovirus A1-E1 open reading frames: ORF1 containing the RdRp domain and ORF2 that contains the coat protein domain. N/A= not applicable. Black squares represent self-identities. 2b: Phylogenetic relationships of statoviruses and other most closely-related viruses were inferred from the RdRp amino acid alignment generated by the Bayesian MCMC method. Internal branch labels indicate the posterior probability, labels with less than 0.7 support are not shown. The monophyletic statovirus clade is highlighted in red. 2c: Magnified image of the statovirus clade from 2b. Internal branches indicate the bootstrap support/posterior probability, labels with less than 0.7 support are not shown. Colored circles represent the mammalian host in which the statovirus genome was identified. (For interpretation of the references to color in this figure legend, the reader is referred to the web version of this article.)

We further examined these datasets for sequencing reads with more distant relationship to statovirus A1. Using tBLASTn we identified reads that upon translation aligned to the amino acid sequence of statovirus A1. From one specimen in cohort 2 we assembled a 4,135 nt contig with > 2.9x coverage that shared 27% amino acid similarity to the ORF1 sequence of statovirus A1 (Fig. 2a). Hereafter, we will use the arbitrarily selected criterion of < 50% amino acid identity in the RdRp domain by BLASTp to differentiate distinct types of statoviruses, delineated by distinct letters; thus, this contig was designated “statovirus B1” (NCBI GenBank accession number KX792980). An additional 36 samples containing contigs of greater than 500 nt that had > 90% nucleotide identity to statovirus B1 were identified from macaque cohorts 2 and 3 by BLASTn. Another distant contig was identified from a human stool sample from a child living in Malawi, and we named this contig “statovirus C1” (NCBI GenBank accession number KX792981). This contig was 3,229 nt in length with > 100x coverage and upon translation to amino acid shared ≤36% identity to ORF1 of both statovirus A1 and statovirus B1 (Fig. 2a). No additional reads from other samples could be aligned to the statovirus C1 contig using BLASTn. Further iteration using the translated amino acid sequences of statovirus B1 and C1 contigs as queries for tBLASTn did not identify any additional divergent contigs in these datasets.

Next, we evaluated public sequencing datasets in the NCBI Sequence Read Archive (SRA) for the presence of statovirus sequences. Our search strategy targeted datasets representing broad ecological niches that included sequencing of RNA (total of 5,457 SRA datasets). Using the statovirus A1, B1, and C1 contigs as reference sequences, we queried datasets by tBLASTn to identify reads that aligned to the translated amino acid sequences. Two SRA datasets were identified that contained reads with similarity to statoviruses that could be assembled into contigs. The first contig was from a *Bos taurus* rumen sample from Beijing, China (SRA accession SRR1604865) (Dai et al., 2015). The assembled contig was 1,875 nt in length with > 3.8x average coverage and the translated amino acid sequence shared ≤33% amino acid identity to the ORF1 sequences of statovirus A1, B1 and C1 and thus was named “statovirus D1” (Fig. 2a; NCBI GenBank accession number KX792982). A second contig named “statovirus E1” (NCBI GenBank accession number KX792983) was assembled from a wild *Mus musculus* stool specimen from Virginia, United States (SRA accession SRR149190) (Phan et al., 2011). The length of this contig was 4,443 nt with > 50x average coverage and it shared ≤35% amino acid identity to the ORF1 sequences of statovirus A1, B1, C1 and D1 (Fig. 2a). No contigs of statovirus A1, B1, or C1 could be assembled from the public metagenomic datasets. Repeating the analysis with statovirus D1 and E1 contigs as the query amino acid sequence did not identify any further reads using our cutoffs for assembly. We did identify three datasets that contained > 5 reads but these had limited similarity (alignment bitscores of < 50) to the RdRp region of statoviruses. Following assembly, contigs lacked the ORF structure characteristic of statoviruses (data not shown). These contigs may represent previously unidentified viruses that contain a RdRp Superfamily II domain.

### 3.3. Analysis of statovirus sequences

To better characterize the novel statovirus sequences, we first defined the RdRp Superfamily domain contained in the statovirus genomes. We queried each representative statovirus genome using Hmmscan with the Pfam database, and all statoviruses had best alignment to the Superfamily II domain (PF00998) with poorly supported alignments to members of Superfamily I and III (Supplementary Table s1). Next, we examined the evolutionary relationship of statoviruses to its most closely-related RNA viruses. Phylogenetic trees were constructed using the Bayesian method to infer the RdRp amino acid alignment of 77 virus sequences (multiple sequence alignment included as supplementary data s1). Statoviruses form a well-supported, monophyletic clade distinct from distantly-related flaviviruses and tombusviruses (Fig. 2b). A magnified view of the statovirus clade is depicted in Fig. 2c.

### 3.4. Genome organization of statoviruses

Statovirus A1, B1, C1, and E1 contigs all share similar predicted genome organizations with only two ORFs that are greater than 600 nt in length (Supplementary Table 2). ORF1 spans most of the genome and contains a domain that shares sequence similarity to the RdRp Superfamily II domain. The length of ORF1 of statovirus A1 (3,885 nt) suggests that it may encode a polyprotein that includes the RdRp, but aside from the RdRp, there was no detectable similarity to any other known viral protein or domain, based on BLAST, NCBI Conserved Domain, Pfam, or Phyre2 (Finn et al., 2016; Marchler-Bauer et al., 2015; Kelley et al., 2015). ORF2 shares an out-of-phase overlap with ORF1 (Fig. 1). Within ORF2, distant structural alignment to the coat protein of tombusviruses was detected by Phyre2 (e-value 0.0038 for statovirus A1; Fig. 1) (Kelley et al., 2015). Of these contigs, the statovirus D1 contig was the shortest (Supplementary Table 2). While it clearly contained a RdRp Superfamily II domain, no reliable inference about the presence of an ORF2 could be made due to the limited sequence length.

To identify viruses with similar genome organization to statoviruses, we also analyzed the annotated genome organization of reference viruses in the ICTV 2015 Virus Taxonomy release and recent publications of novel RNA viruses. Overall, the statovirus genome organization is dissimilar to other reference viruses of the RdRp Superfamily II domain (Fig. 1). We did find two viruses with similar genomes, the Beihai noda-like virus 10 (KX883117) and nudaurelia capensis beta virus (NCBV; NC_001990.1) (Shi et al., 2016; Zeddam et al., 2010). These viral taxa encode genomes that contain two ORFs, a larger ORF that spans most of the genome that contains the RdRp domain, and a smaller, overlapped ORF that contains the coat protein. However, Beihai noda-like virus 10 encodes a RdRp Superfamily I domain and NCBV encodes a RdRp Superfamily III domain. The presence of different RdRp domains in these viruses suggests a very distant relationship to the RdRp domain of statoviruses.

### 3.5. Sequence diversity of statovirus A in two large cohorts of macaques

To define the sequence diversity of statovirus A, we generated a degenerate primer pair that was designed to detect all of the known variants of the statovirus A, by targeting sequences conserved with statovirus C1 (Table 1). We performed RT-PCR with this primer set on samples from macaque cohort 2 and 3. We selected these cohorts because of the high frequency in which statovirus contigs were identified, the relatively large cohort sizes, and the availability of samples in some cases from two timepoints. For cohort 2, 28/120 (23%) samples were positive by RT-PCR for statovirus A. In cohort 3, 20/71 (28%) stool samples were positive by RT-PCR. All RT-PCR amplicons were Sanger sequenced (NCBI GenBank accession numbers KX792984- KX793031).

**Fig. 3:**
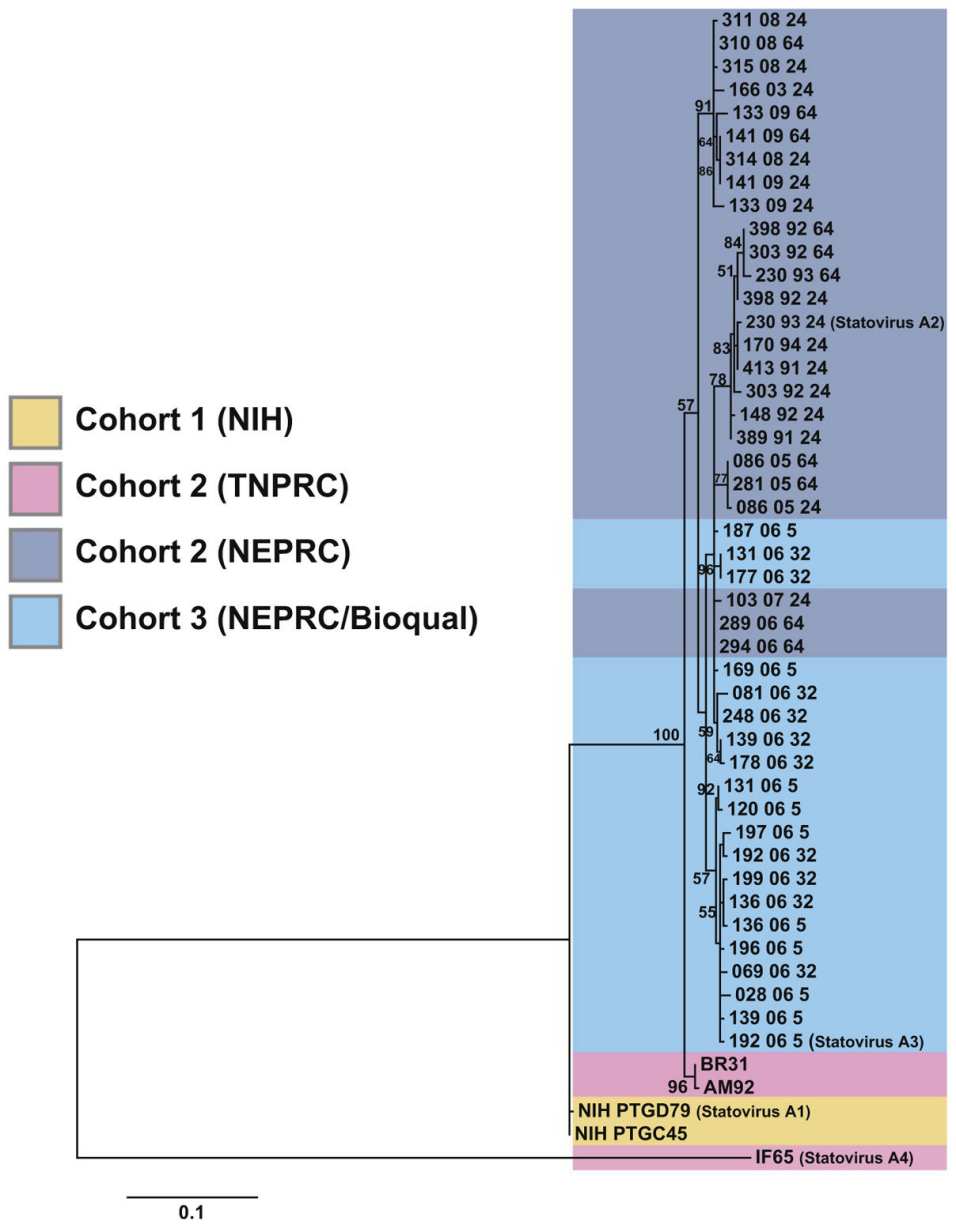
Maximum likelihood phylogenetic tree of statovirus A. Sequenced amplicons from cohorts 2 and 3 and the contigs identified from cohort 1 containing the corresponding region were included in the phylogenetic tree. One hundred bootstraps were performed, and bootstrap scores > 50 are depicted.

The RT-PCR amplicons were subjected to phylogenetic analysis along with the corresponding region of the statovirus contigs from cohort 1 (NCBI GenBank accession number KX793036) to represent the phylogenetic diversity of all three macaque cohorts (Fig. 3). In general, we noted clustering of sequences based on geographical location. Three samples from TNPRC contained statovirus A, and two of the sequenced amplicons forming a well-supported clade. A third amplicon was divergent with only 80% nucleotide identity by BLASTn as compared to all other amplicons, which was designated “statovirus A4” (NCBI GenBank accession number KX792989). Interestingly, all the samples collected from the NEPRC or Bioqual site of cohorts 2 and 3 formed a clade with samples interspersed with each other, despite being part of separate experimental groups. Lastly, most sequenced amplicons were unique, but there were four pairs and one triad of macaques stool samples that contained amplicons with 100% nucleotide identity. The presence of clades based on geography and the presence of many unique amplicons from RT-PCR would suggest against the possibility of a single source of contamination to all of the samples.

### 3.6. Detection of statovirus C1 in the stool of a symptomatic human subject

Statovirus C1 was identified from a human stool sample that was part of a case-control cohort of children diagnosed with and without environmental enteropathy. These children were enrolled longitudinally so multiple stool samples were available from the same subject. The positive stool sample for statovirus C1 originated from a 3.6-year-old female from Malawi who had environmental enteropathy. In the seven days prior to providing the stool sample that contained statovirus C1, she experienced symptoms of fever, cough, and rash, with resolution of the fever and rash prior to the day the stool sample was collected. Notably, she did not have symptoms of diarrhea. NGS of the sample positive for statovirus C1 also identified reads with closest sequence alignment with other viruses including enteroviruses, cardioviruses, circoviruses, picobirnaviruses, anelloviruses, virgaviruses, chloroviruses, and unclassified viruses.

The presence of the statovirus C1 contig was confirmed by RT-PCR and subsequent Sanger sequencing. A total of 2,911 out of 3,229 nucleotides of the assembled NGS contig was confirmed by Sanger sequencing with ≥3x coverage (NCBI GenBank accession number KX792981). There was 99.89% sequence congruence between the Sanger sequenced genome when compared to the assembled contig from MiSeq platform. The 3′ end of the genome was further extended by 32 nt using 3′ RACE to generate a final experimentally confirmed contig of 2943 nt. Using primers AJ0057 and AJ0059 we screened additional stool samples from the index case for the presence of statovirus C1. Specimens were available from days 199, 227, and 328 prior to the positive sample and all tested negative for statovirus C1. The positive stool sample for statovirus C1 was the final sample the index case provided for the study, so we could not assess the duration in which statovirus C1 was detectable. The index case also had a dizygotic twin who was enrolled in the study who did not have environmental enteropathy. He provided stool samples on the same days as the index case and all of his stool samples were statovirus-negative by RT-PCR.

## 4. Discussion

We identified a novel clade of viruses in mammalian GI tract samples from geographically diverse locations. Both phylogenetic analysis and genome organization support the argument that statoviruses should be classified as a distinct taxon separate from the families *Tombusviridae* and *Flaviviridae*. Statoviruses form a well-supported monophyletic clade based on phylogenetic analysis (Fig. 2b). In addition, statoviruses have a unique genome organization that differs from both of these families. Tombusviruses encode 3–5 kb sized genomes with 3–5 ORFs and lack a large open reading frame that spans the entire genome (Fig. 1). All tombusviruses also contain a read-through stop codon which can be suppressed or encode a frame-shift mechanism for translation of the RdRp, which has not been identified in the statovirus genome organization (International Committee on Taxonomy of Viruses, King AMQ, 2012). Statoviruses also lack any detectable sequence similarity or conserved domain resembling the movement proteins found in all tombusviruses, an important protein for facilitation of cell-to-cell spread of plant viruses (International Committee on Taxonomy of Viruses, King AMQ, 2012; Heinlein, 2015; Lucas, 2006). The flavivirus genome structure also contrasts with the genome structure of statoviruses as flaviviruses encode a single ORF that spans a 9.7–12 kb genome and lack an overlapped ORF containing a coat protein sequence (Fig. 1) (International Committee on Taxonomy of Viruses, King AMQ, 2012). In addition, there are no regions within the statovirus genome that share detectable amino acid similarity to the non-structural proteins of flaviviruses.

Whether statoviruses infect mammals, plants, or other organisms within the mammalian gut is currently unclear. The presence of these sequences in datasets acquired from mammalian gut metagenomes raises the possibility that this clade of viruses infects the mammalian GI tract, although we currently do not have experimental data to either support or refute this possibility. Interestingly, no statovirus sequences were detected in public sequencing datasets from bacteria, fungi, plants, unicellular eukaryotic organisms, or environmental samples. A dietary origin of statoviruses is possible as the extensive statovirus sequence diversity within cohorts could reflect a diverse viral population contaminating the food supply.

Statovirus C1 was identified from a human stool sample of a symptomatic young child with fever, cough, and rash. Given the temporal relationship between the symptoms and the detection of this virus, statovirus C1 could be a cause of a non-specific febrile illness of humans. Other viruses associated with disease in humans were also detected from the same stool sample and could be an alternative cause of the patient’s symptoms. Further epidemiologic and seroprevalence studies of statovirus C1 will be required in order to substantiate the possibility of this virus causing symptomatic disease in humans.

Our results also serve to underscore the importance of unbiased analysis of all members of the virome. Novel viruses with distant sequence alignment to known viruses may represent novel viruses within a family or could define a completely distinct family. For instance, in this case, the statoviruses could have easily been dismissed as “plant viruses”. While we have yet to define the host of Statoviruses, it is clear that they are not simply modest variants of known tombusviruses, like beet black scorch virus; rather they form a distinct phylogenetic clade with distinct genomic organization. Furthermore, many viral families such as *Bunyaviridae*, *Rhabdoviridae*, and *Reoviridae* infect a diversity of hosts ranging from plants to humans.

The results herein do raise the possibility that statoviruses are mammalian infecting viruses. Future work would include development of cell culture models and determination of whether serologic responses to statoviruses occur in the putative host species. Finally, additional prevalence studies of these five unique statovirus genomes, as well as determination of whether additional novel statoviruses exist, are needed to define the potential relevance of statoviruses to mammalian health and disease.

## Supplementary Material

1

2

3

## Figures and Tables

**Table 1:** Primers used for sequencing and screening for statovirus A and C. Nucleotide position is based on the statovirus A1 contig or the statovirus C1 contig. Primers AJ0057 and AJ0059 are based on the location in the statovirus A1 contig.

Name	Direction	Sequence	Nucleotide position	Annealing temperature
Sequencing primers of statovirus A2				
AJ0092	Forward	CATATCTCTGGGTTAAGAG	1	51 °C
AJ0001	Reverse	GCAATCAAAGCTGTTAAATAATTC	549	51 °C
AJ0002	Forward	GTCCCAGAATCTTACAATGAC	505	54 °C
AJ0003	Reverse	ATTTTCAATTGTACCAGGAGG	1518	54 °C
AJ0004	Forward	GCTATGATGTTCCCTGGAT	1273	54 °C
AJ0005	Reverse	CAATAACTCACCCATGTACG	2340	54 °C
AJ0006	Forward	AGATATAAGTATTGTTGTTGTTGTG	2260	54 °C
AJ0007	Reverse	GTAAGTTGTCCAATCGAAGTC	3170	54 °C
AJ0008	Forward	GTTTACAATCAGTCAAGTGGG	3116	54 °C
AJ0009	Reverse	TTAAGTGGTCCTGCTCC	4021	54 °C
AJ0010 3′ RACE	Forward	CAAAGTGCTGGACCAAC	3904	54 °C
Poly-T primer for 3′ RACE	Reverse	GGCCACGCGTCGACTAGTACTTTTTTTTTTTTTTTTT	N/A	54 °C
Sequencing primer of statovirus C1				
AJ0068	Forward	CCGAATGATAGTCAGCTACG	298	54 °C
AJ0069	Reverse	GCGATCATCAAAATTCAAGCC	1292	54 °C
AJ0066	Forward	CACGCCGAAGTTTAATGTGG	1175	55 °C
AJ0067	Reverse	CTTTACCGCCCTTTCCTGTC	2114	54 °C
AJ0054	Forward	GGTCTTCTTGCTAAGATATG	2028	53 °C
AJ0064	Reverse	GTCTTTCCACTCTTCGACTG	2960	53 °C
AJ 0066 3′ RACE	Forward	GGAAAGGAATTGAAGTCGC	2835	53 °C
Screening primer for statovirus A and statovirus C1				
AJ0057	Forward	GGYCTICTTGCWAARHTATG	2959	53 °C
AJ0059	Reverse	RTCTKRYCCTCTRCATGGTC	3424	53 °C

N/A = not applicable.

## Appendix A. Supporting information

Supplementary data associated with this article can be found in the online version at doi:10.1016/j.virol.2017.01.010.
