# Supplementary material for: Statoviruses, A novel taxon of RNA viruses present in the gastrointestinal tracts of diverse mammals
Source: Virology. Author manuscript; Available in PMC 2017 Jul 18. (PMC5515247; doi:10.1016/j.virol.2017.01.010)
Supplement: 1 [file NIHMS859465-supplement-1.docx]

Supplementary Table S1: Hmmscan alignment scores of Statovirus sequences to RdRp Superfamily domains. Amino acid sequences of ORF1 were queried against the Pfam protein family database. The alignment length in amino acids, bitscore, and e-value result for each alignment are shown. N/A= No alignment

|  | Superfamily I (RdRp 1) | | | Superfamily II (RdRp 3) | | | Superfamily III (RdRp 2) | | |
| --- | --- | --- | --- | --- | --- | --- | --- | --- | --- |
|  | Alignment length | Bit score | Conditional  E-value | Alignment length | Bit score | Conditional  E-value | Alignment length | Bit score | Conditional  E-value |
| Statovirus A1 | 108 | 18 | 9.3e-08 | 231 | 75 | 4.4e-25 | N/A | N/A | N/A |
| Statovirus B1 | 127 | 10 | 4.5e-05 | 220 | 38 | 1.4e-13 | 118 | 30 | 4.7e-11 |
| Statovirus C1 | 105 | 15 | 6.9e+00 | 248 | 24 | 1.2e-09 | N/A | N/A | N/A |
| Statovirus D1 | 42 | 19 | 7.0e-08 | 213 | 46 | 3.7e-16 | 101 | 15 | 1.3e-06 |
| Statovirus E1 | 111 | 15 | 1.1e-06 | 209 | 31 | 3.1e-11 | 186 | 18 | 2.9e-07 |
