# Supplementary material for: Statoviruses, A novel taxon of RNA viruses present in the gastrointestinal tracts of diverse mammals
Source: Virology. Author manuscript; Available in PMC 2017 Jul 18. (PMC5515247; doi:10.1016/j.virol.2017.01.010)
Supplement: 2 [file NIHMS859465-supplement-2.docx]

Supplementary Table 2: Genome annotation of statoviruses. Nucleotide positions of each ORF, protein domain, and 3’ UTR are included. The first in-frame ATG sequence was selected as the start codon of each ORF. N/A= not available

| Statovirus A1 | | | |
| --- | --- | --- | --- |
|  | Start | End | Length |
| ORF1 | 73 | 3957 | 3885 |
| RdRp domain | 1975 | 2670 | 696 |
| ORF2 | 2979 | 4109 | 1131 |
| Coat protein domain | 3090 | 3632 | 543 |
| 3’ UTR | 4110 | 4158 | 49 |
| Statovirus A2 | | | |
|  | Start | End | Length |
| ORF1 | 54 | 3938 | 3885 |
| RdRp domain | 1950 | 2651 | 702 |
| ORF2 | 2861 | 4090 | 1230 |
| Coat protein domain | 3071 | 3592 | 522 |
| 3’ UTR | 4091 | 4136 | 46 |
| Statovirus A3 | | | |
|  | Start | End | Length |
| ORF1 | 38 | 3922 | 3885 |
| RdRp domain | 1934 | 2674 | 741 |
| ORF2 | 2944 | >3984 | >1041 |
| Coat protein domain | 3055 | 3576 | 522 |
| 3’ UTR | N/A | N/A | N/A |
| Statovirus B1 | | | |
|  | Start | End | Length |
| ORF1 | 134 | 3760 | 3627 |
| RdRp domain | 1904 | 2566 | 663 |
| ORF2 | 2962 | 4098 | 1137 |
| Coat protein domain | 3184 | 3696 | 513 |
| 3’ UTR | 4099 | 4135 | 37 |
| Statovirus C1 | | | |
|  | Start | End | Length |
| ORF1 | 78 | 2897 | 2820 |
| RdRp domain | 699 | 1364 | 666 |
| ORF2 | 1727 | 2827 | 1101 |
| Coat protein domain | 1856 | 2308 | 453 |
| 3’ UTR | 2898 | 2943 | 46 |
| Statovirus D1 | | | |
|  | Start | End | Length |
| ORF1 | 176 | >1875 | >1700 |
| RdRp domain | 884 | 1522 | 639 |
| ORF2 | N/A | N/A | N/A |
| Coat protein domain | N/A | N/A | N/A |
| 3’ UTR | N/A | N/A | N/A |
| Statovirus E1 | | | |
|  | Start | End | Length |
| ORF1 | 506 | >4443 | >3938 |
| RdRp domain | 1859 | 2488 | 630 |
| ORF2 | 2980 | 4197 | 1218 |
| Coat protein domain | 3118 | 3759 | 642 |
| 3’ UTR | N/A | N/A | N/A |
